# Rational surgical neck management in total laryngectomy for advanced stage laryngeal squamous cell carcinomas

**DOI:** 10.1007/s00432-020-03352-1

**Published:** 2020-08-18

**Authors:** Arne Böttcher, Christian S. Betz, Stefan Bartels, Bjoern Schoennagel, Adrian Münscher, Lara Bußmann, Chia-Jung Busch, Steffen Knopke, Eric Bibiza, Nikolaus Möckelmann

**Affiliations:** 1 Department of Otorhinolaryngology, University Medical Center Hamburg-Eppendorf, Martinistraße 52, 20246 Hamburg, Germany; 2 Clinical Cancer Registry, University Cancer Center Hamburg, University Medical Center Hamburg-Eppendorf, Martinistraße 52, 20246 Hamburg, Germany; 3 Department of Diagnostic and Interventional Radiology and Nuclear Medicine, University Medical Center Hamburg-Eppendorf, Martinistraße 52, 20246 Hamburg, Germany; 4 Department of Otorhinolaryngology, Kath. Marienkrankenhaus GmbH, Alfredstraße 9, 22087 Hamburg, Germany; 5 Department of Otorhinolaryngology, Charité – Universitätsmedizin Berlin, Campus Virchow-Klinikum, Augustenburger Platz 1, 13353 Berlin, Germany; 6 Institute of Medical Biometry and Epidemiology, University Medical Center Hamburg-Eppendorf, Martinistraße 52, 20246 Hamburg, Germany

**Keywords:** Total laryngectomy, Neck dissection, Advanced laryngeal cancer, Nodal yield, Level IIB, HNSCC

## Abstract

**Purpose:**

Controversies exist in regard to surgical neck management in total laryngectomies (TL). International guidelines do not sufficiently discriminate neck sides and sublevels, or minimal neck-dissection nodal yield (NY).

**Methods:**

Thirty-seven consecutive primary TL cases from 2009 to 2019 were retrospectively analyzed in terms of local tumor growth using a previously established imaging scheme, metastatic neck involvement, and NY impact on survival.

**Results:**

There was no case of level IIB involvement on any side. For type A and B tumor midline involvement, no positive contralateral lymph nodes were found. Craniocaudal tumor extension correlated with contralateral neck involvement (OR: 1.098, *p* = 0.0493) and showed increased involvement when extending 33 mm (*p* = 0.0134). Using a bilateral NY of ≥ 24 for 5-year overall survival (OS) and ≥ 26 for 5-year disease-free survival (DFS) gave significantly increased rate advantages of 64 and 56%, respectively (both *p* < 0.0001).

**Conclusions:**

This work sheds light on regional metastatic distribution pattern and its influence on TL cases. An NY of* n* ≥ 26 can be considered a desirable benchmark for bilateral selective neck dissections as it leads to improved OS and DFS. Therefore, an omission of distinct neck levels cannot be promoted at this time.

## Introduction

Having experienced a mild increase, especially in women in eastern Germany, over the past 20 years (Tinhofer et al. 2015), laryngeal cancer continues to be the third most common manifestation of head and neck squamous cell carcinoma (HNSCC) with an estimated incidence of 177,000 cases per year worldwide (Bray et al. 2018). For advanced stage laryngeal carcinomas (T3–4a), total laryngectomy (TL) is a safe therapeutic option with a similar quality of life compared to organ preservation approaches (Metreau et al. 2014), and it remains the gold standard for cases with invasion through the thyroid cartilage as it provides an improved median overall survival (OS) of 61 months compared to 39 months with definitive chemoradiation (Grover et al. 2015; Bozec et al. 2020). Recent national guidelines underline the importance of TL as a therapeutic option for advanced stage laryngeal cancers (National Comprehensive Cancer Network 2020; Leitlinienprogramm Onkologie 2020; Jones et al. 2016).

Controversies exist in regard to neck management in TL procedures for advanced laryngeal squamous cell carcinoma (ALSCC). The German guidelines recommend an ipsilateral selective neck dissection (SND) of levels IIA to IV for lateralized T3 glottic cancers and extending treatment to the contralateral neck in cases of midline crossing tumor growth in elective (cN0) interventions (Leitlinienprogramm Onkologie 2020). In contrast, recent United Kingdom (UK) and National Comprehensive Cancer Network (NCCN) guidelines do not refer to tumor midline involvement or to sublevel discrimination for neck-dissection (ND) extent. The NCCN guidelines suggest TL with ipsilateral thyroidectomy as indicated, with pretracheal and ipsilateral paratracheal lymph-node dissection of levels II–IV for the cancer types mentioned above (National Comprehensive Cancer Network 2020). UK guidelines recommend bilateral elective neck treatment of levels II–IV for the same cases (Jones et al. 2016).

Neck dissections do have a significant impact on quality of life of patients suffering from HNSCC (Nibu et al. 2010). The surgical extent of laryngeal cancer treatment has a major influence on morbidity, and may lead to prolonged hospitalization and reduced quality of life (Gourin et al. 2015). Therefore, refinement of neck-dissection procedures during laryngectomy seems to be worthwhile in terms of reduction of operating time, costs, and morbidity.

Recently, we stated that ipsilateral ND nodal yield (NY) apparently does not have a significant impact on OS and disease-free survival (DFS) in TL cases, suggesting a more restrained approach towards the ipsilateral neck (Böttcher et al. 2016), even though an NY of ≥ 18 is generally associated with improved OS and could be used as a prognosticator and quality-of-care marker in HNSCC (de Kort et al. 2019). Furthermore, we proposed a novel computed tomography (CT) scan-based scheme for ALSCC midline involvement which suggests omitting contralateral ND in TL cases with tumors lacking midline involvement (“type A”) (Böttcher et al. 2017).

The aim of this study was to evaluate the reproducibility of those earlier studies with a focus on a midline classification scheme based on combined magnetic resonance imaging (MRI) and CT scans. The impact of the NY on survival should also be determined on a different cohort and, in addition, the importance of sublevel discrimination (particularly levels IIA/B) should be examined.

## Materials and methods

### Ethical statement

This article does not contain any experimental study with human participants performed by any of the authors. No identifying information is included in this article. Written informed consent was obtained from all individuals before surgical intervention. For this type of work, formal consent is not required due to its retrospective nature, according to § 12 HmbKHG (Hamburg hospital law).

Following institutional approval by the Clinical Cancer Registry of the University Cancer Center Hamburg, data were reviewed from all patients with histologically confirmed ALSCC who underwent primary TL with bilateral elective or therapeutic neck dissection for curative intent at the University Medical Center Hamburg-Eppendorf between 2009 and 2019. Using specially trained coordinators, data were obtained from a database management system using GTDS (Gießener Tumordokumentationssystem; https://www.med.uni-giessen.de/akkk/gtds/), which thoroughly documents patients’ features using the original pathology reports. Additionally, a review of patients’ digital records was conducted using our local documentation systems myMedis KIS (Getinge) and Soarian® Clinicals (Cerner). Cases were identified using the German Operation and Procedure Classification System (*Operationen- und Prozedurenschlüssel,* OPS) code 5–303 and the German modification of the International Classification of Disease (ICD-10-GM) for Oncology topography code C32.-. Each patient has been directly treated or examined on a follow-up routine by at least one of the authors.

Nodal yield was calculated as the number of harvested lymph nodes from each single neck (sub-)level as far as conducted by the executing surgeon. Neck levels have been separated intraoperatively in the majority of the cases before the specimens were sent to pathology.

Preoperative clinical staging of the primary tumor and neck was determined using CT scans and MRI. Data were verified by authors A.B. and S.B.

Exclusion criteria included salvage TL, TL after induction chemotherapy, TL due to hypopharyngeal SCC, functional TL, TL for non-SCC tumors, history of chemotherapy/-radiation, history of neck dissection, history of cordectomy > Type I, and multi-level growth.

### Radiological assessment of tumor extension

For assessment of tumor midline involvement, the preoperative radiological imaging was reevaluated in a blinded manner by a consultant from the Department of Diagnostic Radiology (B.S.). The imaging scheme was modified, so it could also be applied to MRI scans (CT = 18, MRI = 18). All examinations were performed using intravenous injection of contrast medium (iodine or gadolinium-chelate). Due to chronic kidney failure in one case, CT was performed without administration of intravenous contrast agent. All examinations were acquired within 3 months prior to surgery. Five examinations were acquired at other radiological institutions. Midline involvement was classified as “type A: clear”, “type B: involved”, “type C: exceeded”, or “type D: bilateral growth/origin side indeterminable” according to the scheme proposed earlier (Böttcher et al. 2017). For type D cases with bilaterally involved necks, the side with higher yield of positive nodes was considered ipsilateral. The craniocaudal tumor extension was evaluated on coronal images and supra-/subglottic dimensions were measured in relation to the vocal cords.

### Statistical analysis

Statistical analysis was performed using SAS software (v9.4; SAS Institute, Cary, NC, USA) and R (v3.6.2.; The R Foundation, https://www.r-project.org/). Statistical significance was set at a level of *α* = 0.05 (*p* < 0.05). Tests for normal distribution of results were performed using the Kolmogorov–Smirnov test. For correlation analysis, Spearman’s rank correlation coefficient (Spearman’s rho, *r*_*s*_) was calculated. Laryngeal midline involvement dependency on regional metastatic spread was tested using Fisher’s exact test and logistic regression analysis. For the latter, midline types were grouped (A + B) and (C + D). The F test was used to calculate the significance of the differences between the variances. The Fisher z transformation was used to calculate the significance of the differences between two correlation coefficients. Differences in survival were calculated from the date of TL to the date of death or last known follow-up (OS) or to the date of first disease recurrence or death from any cause (DFS). Differences in survival were analyzed using univariate regression analysis (generalized Wilcoxon Mantel–Cox log-rank for long-term follow-up) using the Chi-squared (*χ*^2^) statistic. Survival curves were generated using the Kaplan–Meier method.

## Results

From an initial cohort of 103 patients who underwent TL procedures over an 11-year period, 37 (35.9%) were identified for further investigation as they met the rigorous inclusion criteria of primary TL for exclusively ALSCC with glottic involvement. Having a mean age of 65.7 ± 11.7 years, the predominantly male cohort (*n* = 35, 94.6%) presented with an ALSCC originating most frequently in the true vocal folds, and showing transglottic growth (*n* = 29, 78.4%) (Table 1). From available polymerase chain reaction (PCR) and immunohistochemistry (IHC) reports (*n* = 19), one case (5.3%) was identified as a human papillomavirus (HPV)-associated (serotype 16) and p16-positive ALSCC. Besides two missing adjuvant protocol reports, almost two-thirds of all treated patients received adjuvant treatment after TL, either radiotherapy (*n* = 16, 45.7%) or chemoradiation (*n* = 6, 17.1%).

**Table 1:** Patient characteristics (*n* = 37)

	*n* (%)
Age in years at TL (mean = 65.7)	
< 65	15 (40.5)
≥ 65	22 (59.5)
Sex	
Male	35 (94.6)
Female	2 (5.4)
Subsite	
Glottis/transglottic	29 (78.4)
Supraglottis	6 (16.2)
Subglottis	2 (5.4)
pT	
3	20 (54.1)
4a	17 (45.9)
pN*	
0	22 (59.6)
1	2 (5.4)
2a	1 (2.7)
2b	2 (5.4)
2c	4 (10.8)
3a	0 (0)
3b	6 (16.2)
AJCC stage^a^	
III	15 (40.5)
IVA	15 (40.5)
IVB	6 (16.2)
IVC	1 (2.7)
p16^INK4^^a^ (IHC)	
Positive	1 (5.3)
Negative	18 (94.7)
Unknown	18
HPV DNA (PCR)	
Positive	1 (5.3)
Negative	18 (94.7)
Unknown	18
Adjuvant treatment^b^	
None	13 (37.1)
Radiotherapy	16 (45.7)
Chemoradiation	6 (17.1)
Unknown	2

*AJCC*, American Joint Committee on Cancer, *DNA* deoxyribonucleic acid, *HPV* human papillomavirus, *IHC* immunohistochemistry, *PCR* polymerase chain reaction, *TL* total laryngectomy

^a^Adapted from the 8th edition of the AJCC Cancer Staging Manual 2017

^b^Adjuvant protocol reports were only available for 35 patients

Pathology reports revealed a mean bilateral NY of 53.59 ± 28.79 (median: 51). For the ipsilateral NY, a mean of 26.92 ± 14.73 (median: 26) and for the contralateral neck, a mean of 26.40 ± 14.06 (median: 27) was seen, and there was no statistically significant difference between the two neck sides (*p* = 0.878).

**Fig. 1:**
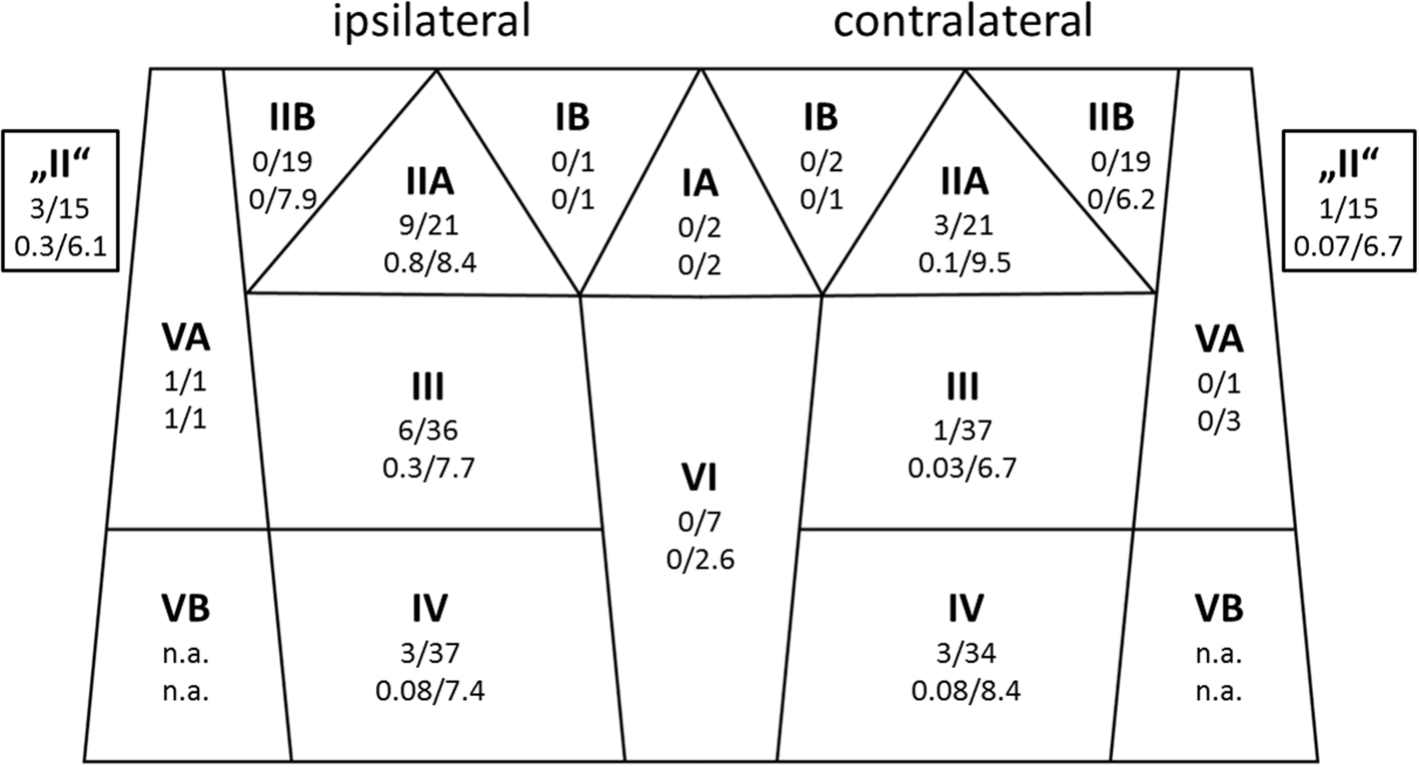
Neck level involvement using a simplified topographical scheme. Bold face: level name; top line: no. cases of positive node(s) from pathology/no. cases dissected; bottom line: no. mean positive nodes/mean nodal yield. Level “II” cases lacked a sublevel distinction into A or B on pathology reports

Based on the pathology reports, 22 patients (59.5%) had a pN0 neck. Seven cases (18.9%) revealed an ipsilateral and 8 (21.6%) a contralateral lymph-node involvement. The cN0 cohort (*n* = 28, 75.7%) experienced an ipsilateral neck involvement in four cases (14.2%) and bilateral neck involvement in three cases (10.7%), which yields an incidence for occult lymph-node metastases of 24.9%. The highest frequencies for regional metastatic manifestations were found for ipsilateral levels IIA (42.9%), “II” (20.0%), III (16.7%), and IV (8.1%) (Fig. 1). In cN0 cases, ipsilateral level IV was involved in two cases (7.1%). On the contralateral neck, the most frequently involved levels were IIA (14.3%), IV (8.8%), “II” (6.7%), and III (2.7%). None of the cases showed level IIB involvement, either ipsilateral or contralateral. The same applied to levels IA, IB bilaterally, contralateral VA, and level VI.

**Fig. 2:**
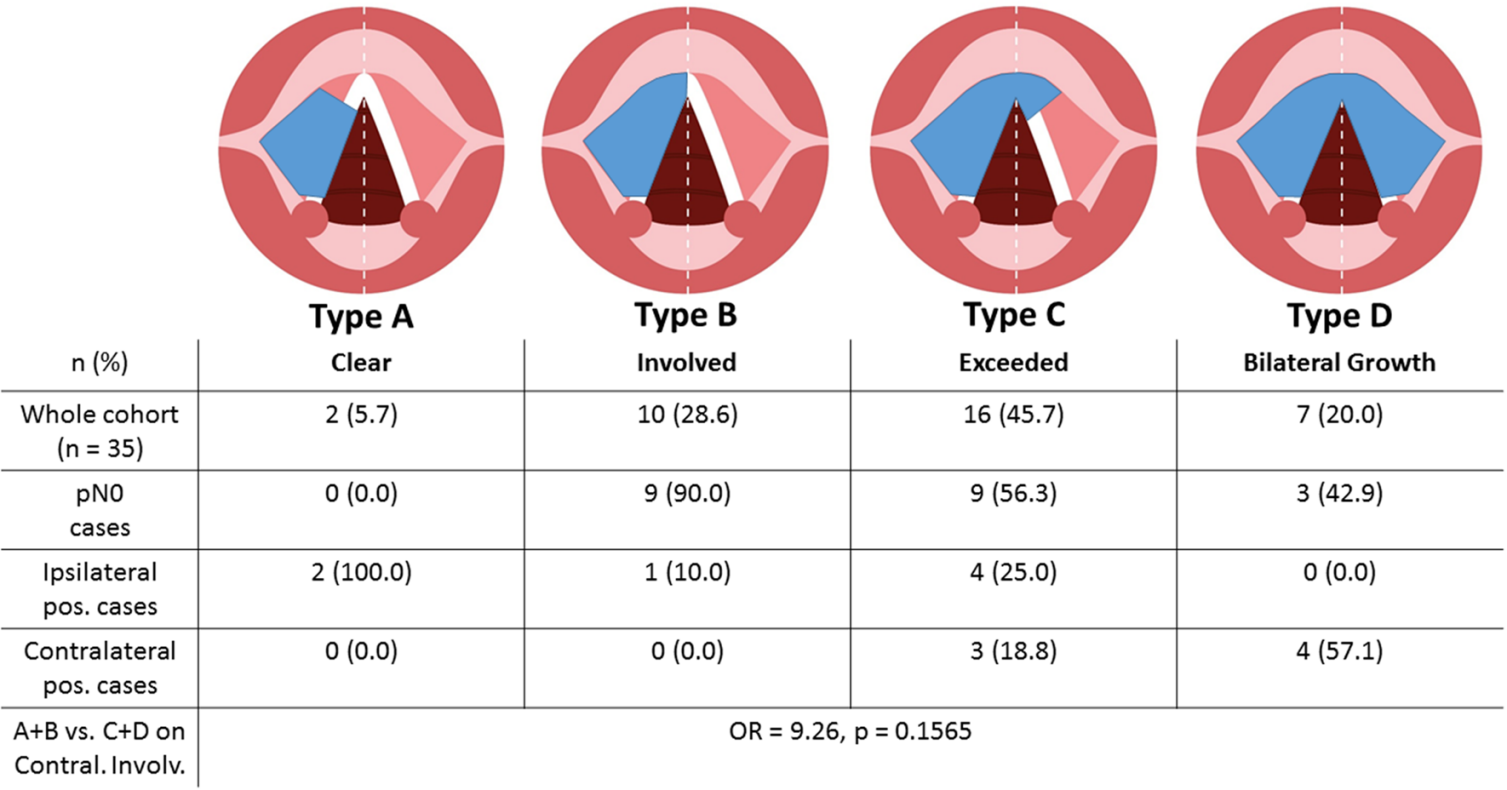
Midline involvement scheme* and case distribution. For types A and B, no contralateral neck involvement was detected on pathology. A significant uneven distribution for midline type and contralateral involvement became evident (*p* = 0.025). For grouped imaging-based midline types, a risk of 4.0% for contralateral involvement in type A + B and 37.14% for type C + D cases was calculated (*p* = 0.1565). *According to Ref. (Böttcher et al. 2017)

The cohort with available imaging consisted of 15 patients (42.9%) who underwent preoperative CT scans and 20 (57.1%) who underwent MRI scans before TL. The majority (*n* = 16, 45.7%) presented with a type C midline involvement on imaging (Fig. 2). Positive contralateral lymph-node(s) cases (*n* = 7, 20.0%) were only seen for type C and type D. Concerning midline involvement type, the Kolmogorov–Smirnov test of normality showed non-normally distributed data (*D* = 0.253, *p* = 0.0181). In rank correlation analysis via Spearman’s rho, a significant correlation was seen between the midline involvement type and positive contralateral lymph nodes (*r*_*s*_ = 0.490, *p* = 0.0028), whereas neither pN status of the 7th and 8th AJCC edition nor overall numbers of positive lymph nodes on histology showed a correlation (*r*_*s*_ < 0.31, *p* > 0.06). A significant uneven distribution for midline type and contralateral involvement became evident (*p* = 0.025). When comparing type A + B against type C + D for contralateral lymph-node involvement on logistic regression analysis as a sensitivity analysis, a non-significant odds ratio (OR) of 9.26 (*p* = 0.1565) was calculated. Analysis resulted in an estimated risk of 4.0% for contralateral involvement in type A + B and 37.14% for type C + D cases.

The craniocaudal tumor extension ranged from 8.0 to 96.0 mm (mean = 34.4 ± 16.9 mm). Based on vocal process level, supraglottic expansion reached a maximum of 47.0 mm (mean = 17.2 ± 11.5 mm), and subglottic growth reached a maximum of 65.0 mm (mean = 16.6 ± 13.1 mm). There was a significant correlation between the appearance of bilateral/contralateral regional metastases and craniocaudal extension (*r*_*s*_ = 0.392, *p* = 0.0242), but not for supraglottic (*r*_*s*_ = 0.306, *p* = 0.0832) or subglottic tumor extension (*r*_*s*_ = 0.1652, *p* = 0.3596). For the contralateral positive node(s) cases, there was a mean craniocaudal extension of 51.5 ± 21.5 mm, significantly higher than mean craniocaudal extension in the contralateral negative node(s) cases (30.6 ± 12.5 mm; *p* = 0.0311). The mean supraglottic extension of the contralateral positive node(s) cases was 26.5 ± 15.0 mm, being not significantly different from the contralateral negative node(s) group (17.7 ± 11.1 mm; *p* = 0.1370).

**Fig. 3:**
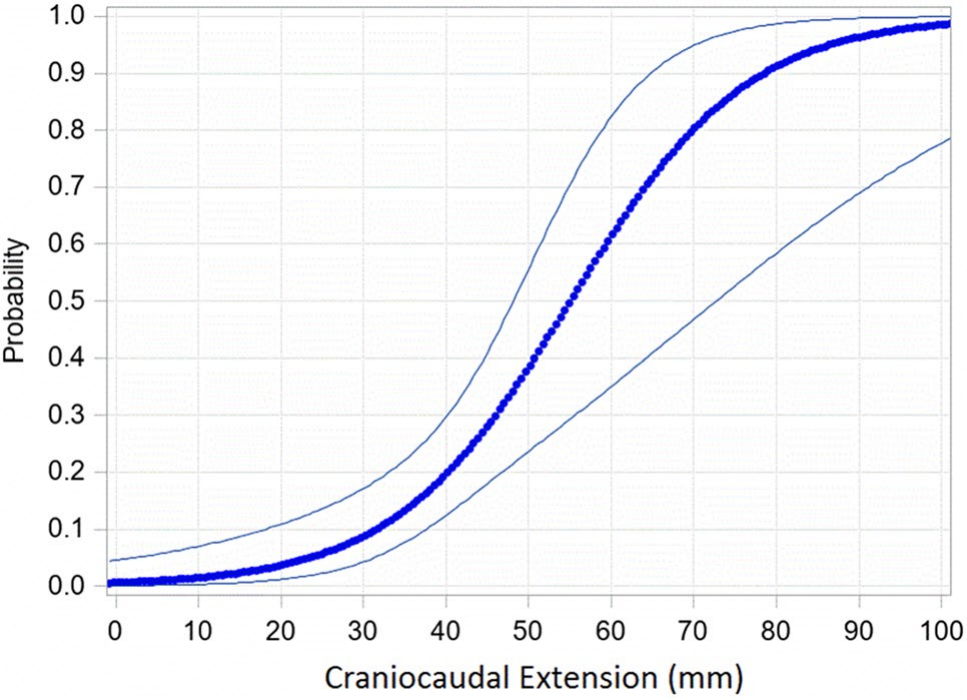
Risk estimation for contralateral lymph-node involvement depending on craniocaudal tumor extension. Craniocaudal extension exerted a significant influence on the appearance of contralateral lymph-node development on histology with an odds ratio of 1.098 (CI 1.000–1.205, *p* = 0.0493) per mm of growth

When comparing the correlation coefficients of the tumor midline involvement type and the craniocaudal tumor extension for a correlation with positive contralateral lymph node(s), no statistically significant difference was found (*z* = 0.48, *p* = 0.6312). On logistic regression analysis, the craniocaudal extension exerted a significant influence on contralateral lymph-node development, showing an OR of 1.098 (CI: 1.000–1.205, *p* = 0.0493) (Fig. 3). This resulted in an estimated risk increase of 9.8% per millimeter of craniocaudal tumor extension. On explorative work-up, a cut-off of 33 mm was found that if exceeded, predicts a significant increase in contralateral metastatic spread (*p* = 0.0134).

### Survival analysis

The whole investigated cohort had a 5-year OS rate of 57% [47% on disease-free survival (DFS)] and an estimated median OS of 62.8 months (27.9 months on DFS) calculated from the date of TL. From the date of diagnosis, a 5-year OS rate of 60% and median OS of 107.9 months was calculated.

**Fig. 4:**
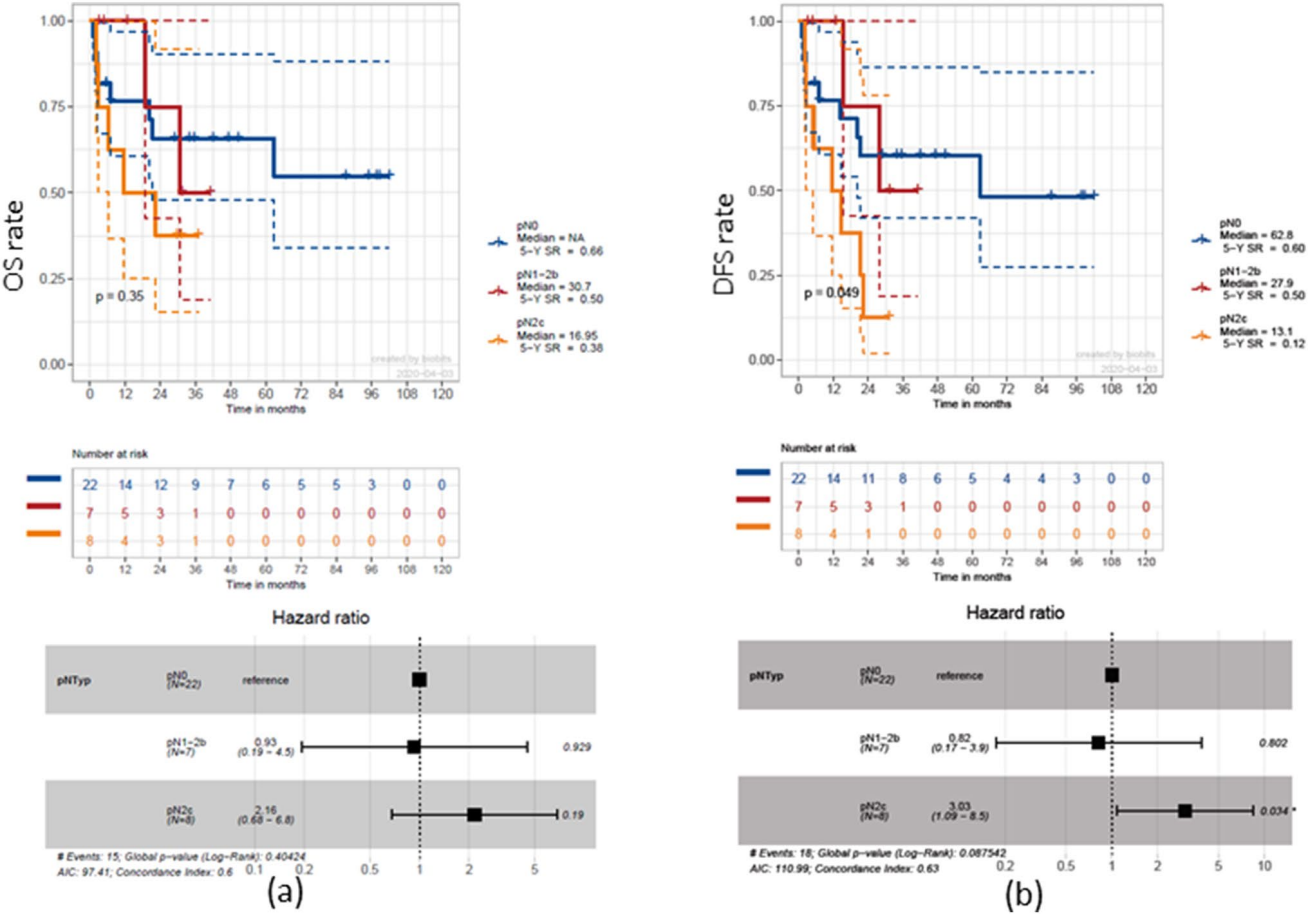
Survival estimation with regard to regional lymph-node involvement. Kaplan–Meier curves depicting overall survival (OS) (**a**) and disease-free survival (DFS) (**b**) for pN0 necks compared to ipsilateral and bilateral pN + necks. There is a significant median DFS advantage of 49.7 months for pN0 necks compared to bilateral neck involvement and 34.9 months for pN0 necks compared to ipsilateral neck involvement (*p* = 0.049)

The presence of regional lymph-node metastases decreased, but not statistically significantly (*p* = 0.35), the 5-year OS rate from 66% for pN0 necks to 50% for ipsilateral pN + necks [hazard ratio (HR) 0.93, *p* = 0.93], and to 38% in bilateral pN + necks (HR 2.16, *p* = 0.19), resulting in a reduced median OS of 16.95 months (bilateral pN +) compared to 30.7 months (ipsilateral pN +) (Fig. 4a). Significant effects of pathologically confirmed positive lymph nodes on neck specimens were shown for DFS (*p* = 0.049). Bilateral lymph-node involvement led to a significantly decreased 5-year DSF rate of 12% compared to 60% for pN0 necks (HR 3.03, *p* = 0.034) (Fig. 4b).

**Fig. 5:**
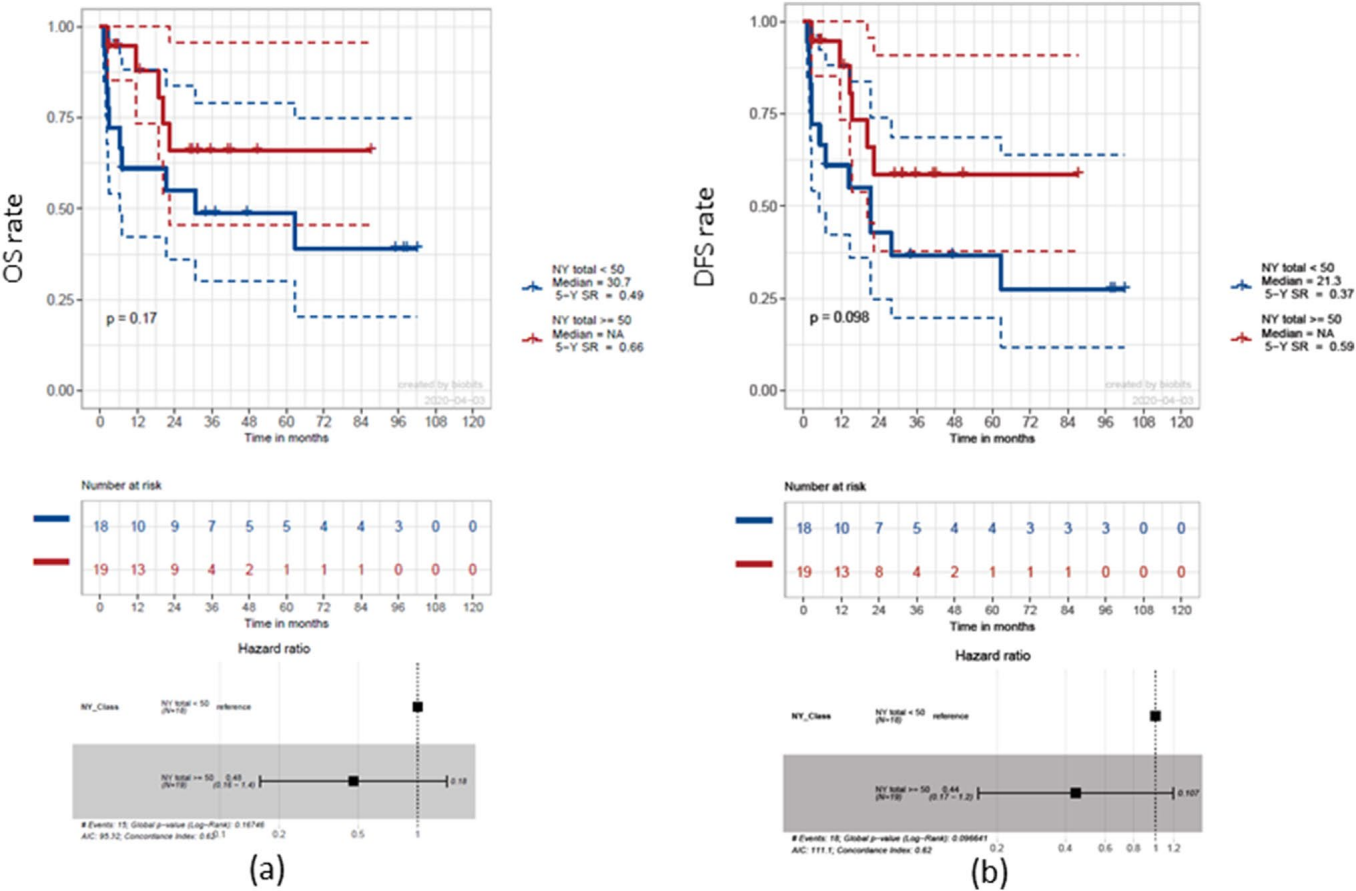
Survival estimation with regard to neck-dissection nodal yield. Kaplan–Meier curves depicting overall survival (OS) (**a**) and disease-free survival (DFS) (**b**) for a nodal yield cut-off at *n* < / ≥ 50, including pN0 cases. Trends towards advantages for a NY ≥ 50 in 5-year OS and DFS rates are evident, but lack statistical significance (17%, *p* = 0.17, and 22%, *p* = 0.098, respectively)

There was no significant effect on OS (*p* = 0.88) or on DFS (*p* = 0.86) for the four different midline types. After calculating the median bilateral NY of *n* = 51, the whole cohort was divided into two groups (*n* < 50 and *n* ≥ 50). A statistically not significant (*p* = 0.17) 5-year OS rate advantage of 17% for a nodal yield of *n* ≥ 50 became evident (HR 0.48, *p* = 0.18) (Fig. 5a). For DFS, a similar not significant effect of that NY cut-off could be detected (HR 0.44, *p* = 0.098) (Fig. 5b).

**Fig. 6:**
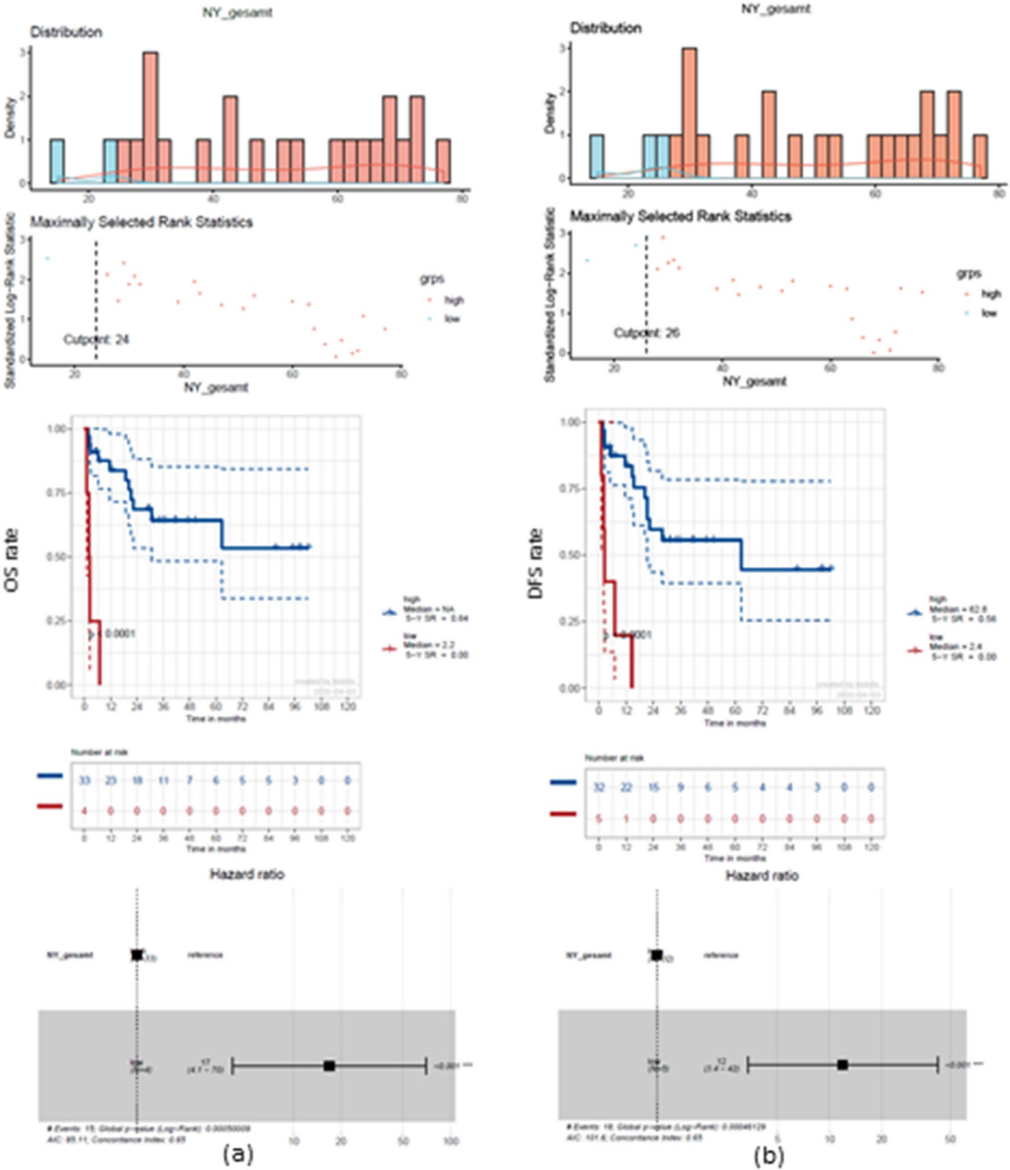
Survival estimation with regard to optimized NY. Cut-off point calculation of *n* = 24 for overall survival (OS) (**a**) and *n* = 26 for disease-free survival (DFS) (**b**). Significant survival advantages are evident leading to an increased median DFS of 62.8 months for a NY > 26 compared to 2.4 months for a NY < 26 (*p* < 0.0001)

To determine a significant NY, optimized cut-off points of *n* = 24 for OS and *n* = 26 for DFS were calculated (Fig. 6). Thus, a 5-year OS rate advantage of 64% (HR 17, *p* < 0.0001) and a 5-year DFS rate advantage of 56% became evident (HR 12, *p* < 0.0001).

## Discussion

This study intended to shed light on regional metastatic distribution patterns for TL cases, including a reevaluation of earlier results, and we were able to show significant influences from tumor growth and surgical neck management. Since Crile first published his series of cervical lymph-node dissection 114 years ago (Silver et al. 2007), there has been an ongoing debate on surgical technique, extent, and clinical benefit of ND for HNSCC (Coskun et al. 2015; Vahl and Hoffmann 2019). Representing a risk for morbidities such as hematoma (9.5%), seroma (5.0%), bleeding (4.8%) (Möckelmann et al. 2015), wound infections (< 5%), chylous fistulas (2%) (Balm et al. 2005), cranial nerve (CN) affection [CN XI 5–20% (Gane et al. 2017), and the marginal mandibular branch of CN VII 7% (Moller and Sorensen 2012)], ND is subject to investigations in terms of reducing the extent from classic modified radical neck dissection ((M)RND) to SND procedures (Teymoortash and Werner 2013). Recently, SND was found to be safe and feasible even in cN + cases (Lopez et al. 2020; Givi et al. 2012). Preservation of levels IIB and IV in laryngeal squamous cell carcinomas with cN0 necks was suggested by Ferlito (Ferlito et al. 2007). Furthermore, it was stated that SND of levels IIA and III would be sufficient for elective neck treatment in glottic and supraglottic carcinoma (cT2–4, cN0) (Ferlito et al. 2008). A major review from 2013 including 609 elective ND showed a probability of 1.7% for level IIB involvement in laryngeal cancers and suggested omitting level IIB dissection in cN0 cases. In pN + necks, 11.4% of all dissected cases showed a level IIB involvement with 80% ipsilateral manifestation (Gross et al. 2013). Unfortunately, that study lacked a detailed tumor staging and description of subsite localization. In another retrospective analysis of 78 patients undergoing both primary TL and TL for recurrences, a selective ND for levels IIA and III, sparing levels IIB and VI in elective cases (cN0), was suggested to be “ideal” (Riviere et al. 2019). A recent randomized-controlled clinical trial has shown that omitting level IIB in elective SND is safe and is accompanied by decreased shoulder function impairment and increased quality of life, while a significant reduction in operating time was also observed. In that study, only one case of laryngeal squamous cell carcinoma was included in the ‘not dissected’ IIB group (Dziegielewski et al. 2019). Similar conclusions were made by a Japanese group who concluded that level IIB dissection in HNSCC was only necessary in cases where preoperative examination revealed multi-level or level IIA metastases or suspected level IIB metastases (Hosokawa et al. 2019). Additionally, level IIB sparing SND seemed to be oncologically coequal, at least in oral cavity cancer (Pandey et al. 2018). Our data support at least the rate of level IIB involvement as it was found to be 0.0%. The lack of clear sublevel designation led to a notable amount of level “II” cases, which presumably included foremost level IIA, and this would have introduced a level of bias in our indicated results.

Data concerning contralateral involvement of lymph nodes in ALSCC are rare (Hamoir et al. 2014). One publication from 1992 specifically dealt with this topic, but unfortunately lacked implications for daily practical routine (Marks et al. 1992). In that study, contralateral regional metastases were found in 4% of all ALSCC (Marks et al. 1992), which is noticeably less than in this study (18.9%) and our earlier work (10.3%) (Böttcher et al. 2017). This difference in detected contralateral lymph nodes might be due to different surgical ND techniques (Lörincz et al. 2016) used in two different centers and consecutive distinctly higher mean ipsilateral NY (*n* = 26.9 in the recent cohort compared to *n* = 18.7 in the earlier one (Böttcher et al. 2016). This work has again shown that contralateral neck involvement is dependent on tumor midline involvement, but, furthermore, is significantly dependent on craniocaudal tumor extension. In addition, the proposed preoperative imaging scheme might be suitable for estimating a certain risk for contralateral cervical spread. We also strongly recommend examining the local craniocaudal extension. Whether omitting one (contralateral) side in neck dissection is oncologically safe must be reevaluated in a study with a larger sample size or in a randomized-controlled trial.

In concordance with our results, a recent retrospective SEER (surveillance, epidemiology, and end results program, National Cancer Institute) database analysis considered an apparently bilateral nodal yield cut-off of *n* > 50 as an independent prognosticator for overall survival (HR: 0.794, *p* = 0.006) using a dataset of partial laryngectomy and TL patients (Zhu et al. 2020). The lack of a statistically significant NY cut-off at *n* = 50 in our work might be attributable to the low sample size of *n* = 37. This also applies to the calculated optimized NY cut-offs of *n* = 24 for OS and *n* = 26 for DFS and the consequent uneven numerical distribution within the groups being compared. For HNSCC, several earlier publications have suggested that a higher nodal yield is advantageous in terms of OS and DFS in HNSCC (de Kort et al. 2019; Ebrahimi et al. 2012; Divi et al. 2016; Pou et al. 2017). Our earlier results of ipsilateral NY lacking impact on survival in TL cases (Böttcher et al. 2016) could not be verified. This might be due to the earlier timespan (2002–2014), the lower mean nodal count of *n* = 18.7 (not reaching the current mean/ipsilateral count of *n* = 26.9), and the lack of sufficient adjuvant treatment protocols. The estimated 5-year OS rates of 60% (from date of first diagnosis) and 57% (from date of TL) for the entire cohort are notably higher than stated in other earlier studies [51.1% (Zhu et al. 2020), 48% (Sullivan et al. 2019), 40% (McGuire et al. 2019), and 32.1% (Böttcher et al. 2016)], which was not solely attributable to NY count.

Some reports have indicated that, in the early stage oral cavity carcinoma, elective SND is superior to a watchful waiting strategy in terms of overall survival and regional recurrence (Ibrahim et al. 2020; Cai et al. 2019). This fact should not be underestimated and survival outcome should not be jeopardized when considering surgical refinement in terms of reducing the extent of dissection. In contrast, several previous retrospective studies showed no significant influence of elective neck dissection (END) on survival in advanced laryngeal cancers (Ketterer et al. 2020; Shi et al. 2019; Kennedy et al. 2017; Djordjevic et al. 2016; Canis et al. 2012). For salvage TL cases, END is not generally recommended, because, although it reduces the rate of regional recurrence, it does not provide a survival benefit as stated in a recent meta-analysis (Davies-Husband et al. 2020).

In 1994, Weiss et al. considered a risk of less than 20% for regional spread in cN0 necks sufficient for observation in primary HNSCC, not differentiating localization, staging, or procedures (Weiss et al. 1994). According to this reference, omission of END (in cN0 cases) should have been a valid option for our cohort. At an incidence of 24.9% occult regional metastases, this would have represented an under-treatment for the same amount of patients. For ipsilateral level IV, an incidence of 7.1% was seen in our cN0 cohort, slightly higher than in another study which reported 3.9% for level IV metastases in ALSCC (Furtado de Araujo Neto et al. 2014).

A recent work suggested that concurrent neck dissection was not associated with increased morbidity in TL procedures (Xiao et al. 2019), which leaves the authors of this study in some doubt when recalling the rates of postoperative complications detected in earlier studies mentioned above including a randomized clinical trial (Dziegielewski et al. 2019).

The imaging assessment was subject to a certain bias as five of the CT/MRI scans were made at other radiologic institutes and have been imported into our Picture Archiving and Communication System (PACS). The scans lacked contrast enhancement in one case, and had a slice thickness ranging from 1 to 5 mm. Additionally, three cases only received CT scans of the thorax/abdomen neglecting areas where the tumor dimensions were not distinctively definable. Nevertheless, according to our findings, imaging checklists should be developed and implemented similar to the novel European Laryngological Society proposal for laryngeal carcinomas before transoral laser microsurgery (Chiesa-Estomba et al. 2020). Therefore, the results of this study offer a basis for further investigations.

### Strengths and limitations

The limitations of this study include its retrospective nature, limited sample size, inconsistent neck level designation, lack of histopathological work-up guidelines for neck samples, and the surgeons’ arbitrary approaches to the neck, at least in cases before 2011, after which time a consistent surgical technique was performed on a regular basis in our institution (Lörincz et al. 2016). The cut-off estimation for NY and craniocaudal tumor extension is subject to a data-driven cut point bias as it was determined on an exploratory basis, but this was necessary due to the limited sample size on which a cut-off calculation resulted in unreasonable figures. The strengths of this study are foremost its restrictively condensed cohort with a small corridor of clinical features and, additionally, its reevaluation characteristic as it was implemented as an inspection of earlier results.

## Conclusions

For rational neck management during TL procedures, regional level IIB involvement is not present, either unilaterally or bilaterally, in ALSCC. While there is an overall incidence of up to 18.9% for contralateral lymph -node involvement, using our proposed midline classification scheme, contralateral regional metastases are not likely to appear (*n* = 0, calculated risk = 4.0%) for type A and B growth patterns. Additionally, craniocaudal tumor extension should be taken into account when addressing the contralateral neck as it is significantly associated with the risk for contralateral metastatic lymph nodes. Occult regional metastases were present in 24.9% of cases.

A bilateral NY of ≥ 26 can be considered a desirable benchmark for SND in TL procedures for ALSCC as it leads to a significant advantage in OS and DFS.

Based on these results, an omission of any of the generally promoted levels (II–IV) or sublevels during SND cannot be suggested at this time, as this would jeopardize survival due to the consequently decreased NY. Prospective randomized clinical trials should focus on survival and morbidity rates when investigating ND extent.
